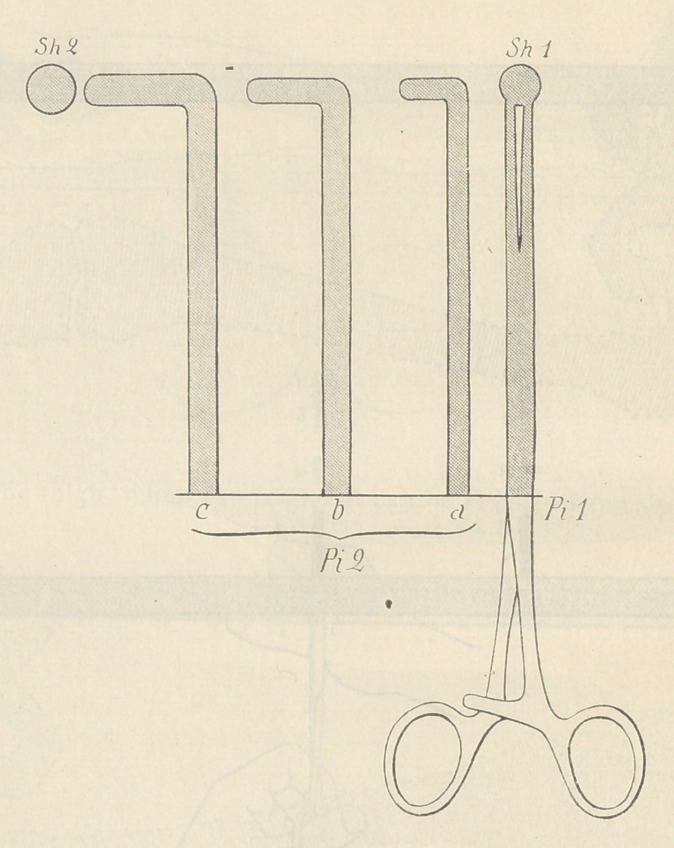# Method of Localization and Extraction of Projectiles by the Simultaneous Use of Two X-Ray Tubes

**Published:** 1918

**Authors:** 


**Method of Localization and Extraction of Projectiles by the Simultaneous Use of Two X-Ray Tubes.** By Dr. de Rio Branco. Trans. and Abs. from the *Bull. et Mém. de la Soc. de Chir. de Paris,* March 26, 1918.

The author projects simultaneously on his screen the shadows of the projectile, obtained in one case by a tube producing vertical rays, and in the other by one producing oblique rays. By regulating the tubes, both rays can be made to project the shadow of the missile to be removed.

This method if of great value as a guide in directing forceps for the extraction of a foreign body. If the prolongation of the shadow of the instrument in both projections passes through the foreign body, the direction of the instrument is seen to be correct. If, however, the prolongation of the shadow of the instrument in one or both of the projections fails to pass through the foreign body, the direction of the instrument in one or both of the two planes is seen to be incorrect.

If, for instance, the operator is trying to remove a shrapnel fragment in the thigh, when the fragment can be reached from a plane perpendicular to that of the rays, the forceps should be placed in contact with the surface of the thigh. Two shadows of the forceps will then appear on the screen corresponding to the two shadows of the missile. If the prolongation of the shadow of the forceps fails in either or both cases to pass through the shadow of the foreign body, the shadows may easily be made to take the right directions by moving the forceps until the correspondence is correct. Whether the penetration is horizontal or vertical, the principle remains the same, and the shadows of the foreign body should be made to lie in the line of prolongation of the direction of the instrument.

This method is also useful in determining the depth of the foreign body in simple cases. For this purpose a mark is placed on the skin at the point where the shadow of the end of the forceps produced by the vertical rays coincides with the shadow of the foreign body. Another mark is then made at the point on the skin where the end of the forceps must rest when the shadows produced by it, if prolonged, pass through the shadows from the foreign body. The depth of the body may then be estimated in the vertical and transversal directions.

## Figures and Tables

**Figure f1:**
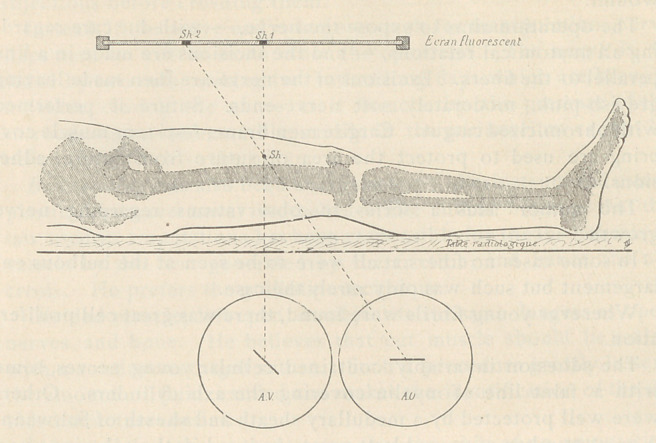


**Figure f2:**
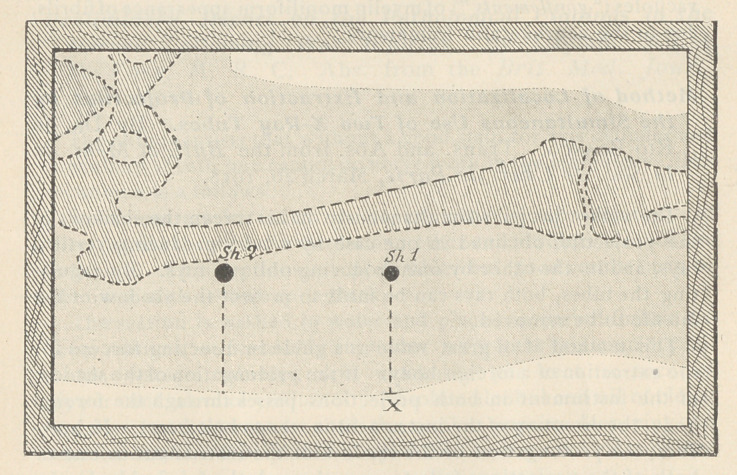


**Figure f3:**
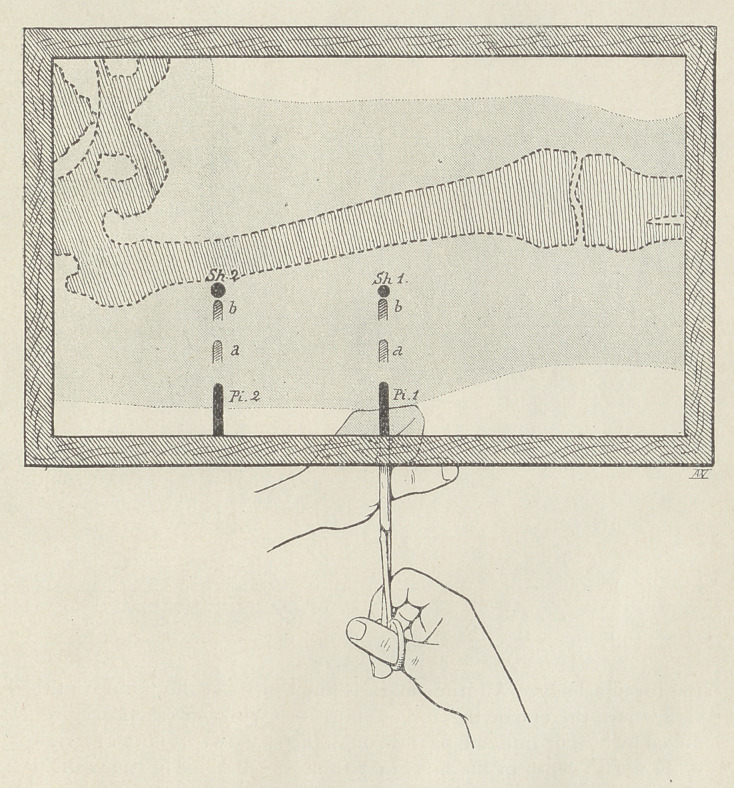


**Figure f4:**